# A Case of Separation of the Symphysis Pubis during Labor

**Published:** 1884-12

**Authors:** E. F. Eldridge


### ARTICLE IV.

### A CASE OF SEPARATION OF THE SYMPHYSIS PUBIS DURING LABOR. *By* E. F. ELDRIDGE, M. D.

The 7th of March last I was called to see a Mrs. D., whom I found to be suffering from prolapse of the rectum. After some little difficulty, I succeeded in replacing the bowel, and as I had noticed that the patient had been unable to walk for some time without the aid of two canes, I took occasion to examine the condition of the pelvis. I found her pregnant, at nearly full term; the abdomen was enormously distended, the wall of the vagina relaxed and partially prolapsed, the symphysis pubis was separted three-quarters of an inch, and the bones at the sacro-iliac synchondrosis quite movable.

She told me that the inability to walk had gradually come on; that she could feel her hips move up and down when she stepped, and that she felt as though she was being pried apart. Attended her in her confinement, which took place a week later; the normal pains came on, and the contractions were strong and regular.

The presentation was normal and in the first position, but progress was slow, the labor lasting seven hours.

The child was a male, weighing 10½ pounds, and looked as though it was at least a month old; the head was large, the fontenells nearly closed and the skull remarkably ossified.

During the passage of the head through the outlet of the pelvis, the symphyses separated one and a quarter inches, so that I could place my two fingers between them.

She made a good recovery, the bones returned to their normal position, and finally united as firmly as before; locomotion is perfect. She told me that she was troubled the same way at her last confinement, but was not so bad.

